# 374. Need to Improve Minority Representation through COVID-19 Community Research Partnership

**DOI:** 10.1093/ofid/ofab466.575

**Published:** 2021-12-04

**Authors:** Keerti Dantuluri, Whitney Rossman, Lauren C Lu, Connell O Dunn, Anna M Harris, Timothy Hetherington, Jennifer Priem, Amina Ahmed, Amina Ahmed

**Affiliations:** 1 Levine Children’s Hospital at Atrium Health, Charlotte, North Carolina; 2 Atrium Health, Charlotte, North Carolina

## Abstract

**Background:**

Minorities are often unrepresented in research, which limits equity in healthcare advances. The racial and ethnic disparities in outcomes of individuals infected with COVID-19 highlight the importance of inclusivity in research to improve public health measures.

**Methods:**

We performed a descriptive analysis of the racial and ethnic distribution of children enrolled in our COVID-19 Community Research Partnership (CRP) study, a syndromic and serological surveillance study of children aged 2 – 17 years receiving care at three healthcare systems spanning North and South Carolina. Syndromic surveillance involved daily symptom reporting using a web-based monitoring application. Participants consenting to serological surveillance were mailed at-home tests sampling finger prick capillary blood. In-person and electronic recruitment efforts were conducted in English and Spanish. At one of the study sites, we compared the racial/ethnic distribution of enrolled children to the racial/ethnic distribution of all children who received care at the same site during the same timeframe. We compared the racial/ethnic distribution of participants who ultimately submitted samples for serological testing compared to those who consented to serologic testing.

**Results:**

At total of1630 children were enrolled from April 2, 2021 – June 8, 2021. Most children were > 5 years old, 50.2% were female, and 88.5% were from mostly urban counties (Table 1). Of enrolled children, 4.2% were Hispanic, 8.2% were black, and 81.6% were white (Table 2). Among 135,355 unique children who received care at the institution during the same time, 12.4% were Hispanic, 23.0% were black, and 63.1% were white. Of 1552 participants who consented to serologic testing, 4.4% were Hispanic, 8.1% were black, and 81.8% were white (Table 3). To date, 242 children submitted serologic samples; 4.1% were Hispanic, 5.0% were black, and 85.5% were white.

Table 1. Characteristics of enrolled children in COVID-19 surveillance study

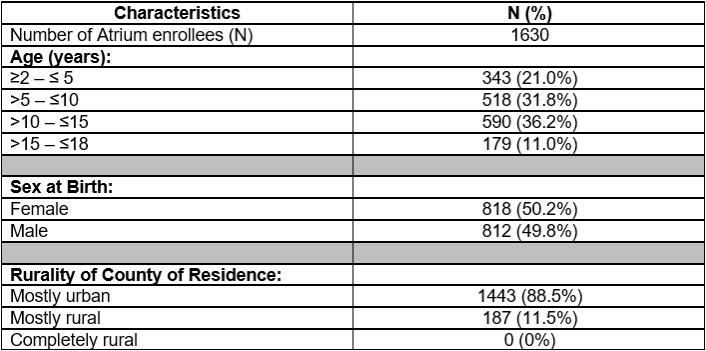

Table 2. Racial and Ethnic distribution of children enrolled in the study compared to target population

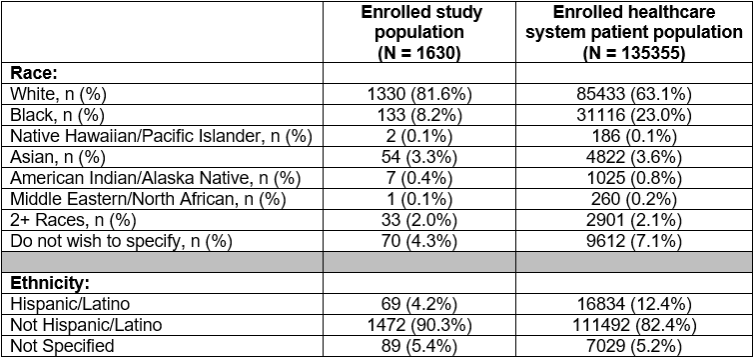

Table 3. Racial and ethnic distribution of children who participated in serology testing

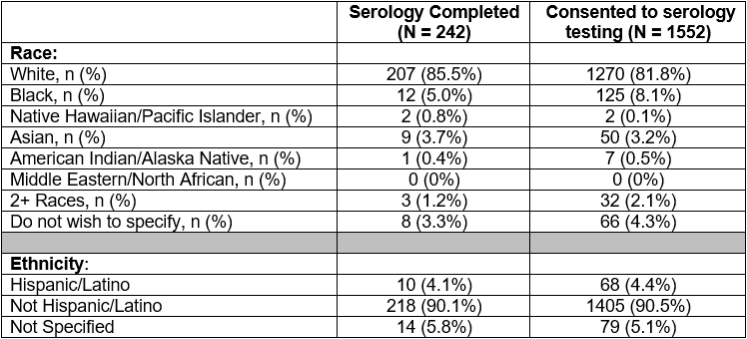

**Conclusion:**

Despite efforts to recruit a diverse group of children, the proportion of minorities enrolled in our COVID-19 surveillance study underrepresents the targeted population. Ongoing efforts will work to identify barriers and facilitators to research participation among minority families.

**Disclosures:**

**Amina Ahmed, MD**, Nothing to disclose

